# Modifications in Carbon and Nitrogen Metabolites of *Vigna unguiculata* L. Seed Organs Induced by Different Priming Treatments

**DOI:** 10.3390/plants14203218

**Published:** 2025-10-20

**Authors:** Lilya Boucelha, Réda Djebbar, Sabrina Gueridi, Othmane Merah

**Affiliations:** 1 Laboratory of Biology and Physiology of Organisms (LBPO), Faculty of Biological Sciences, University Houari Boumediene of Sciences and Technology (USTHB), BP 32 Bab Ezzouar, Algiers 16111, Algeria; liliaboucelha@yahoo.fr (L.B.); sgueridi@gmail.com (S.G.); 2 Laboratory of Agro-Industrial Chemistry (LCA), University of Toulouse, 31030 Toulouse, France; 3 Department of Biological Engineering, University Institute of Technology, University of Toulouse, 32000 Auch, France

**Keywords:** germination, double hydropriming, metabolism, seed, embryo, cowpea

## Abstract

Seed priming has become a promising technique in agriculture and crop-stress management. Several authors have shown that the positive effects of seed priming are associated with various metabolic, physiological, and biochemical modifications (enzyme activation, membrane repair, initiation of DNA/RNA, and protein synthesis) that enhance the speed, uniformity, and vigor of germination. However, the mechanisms underlying seed priming are not yet well understood. The aim of our work was to study the quantitative and qualitative metabolic changes in the embryonic axes (radicle and plumule) and cotyledons of *Vigna unguiculata* (L.) Walp. Seeds were subjected to osmopriming with polyethylene glycol (PEG), simple hydropriming, and double hydropriming (a novel treatment). Results indicated that all types of priming, particularly double hydropriming, strongly stimulated the hydrolysis of protein and carbohydrate reserves. This resulted in a decrease in soluble proteins and starch contents and an increase in amino acids and soluble sugars contents. Moreover, the priming promoted the biosynthesis of osmolytes such as proline and induced qualitative changes in the composition of amino acids and soluble sugars. These biochemical changes depend on the organ and treatment method applied to the seeds. It is worth noting that double hydropriming induces metabolic modifications to a greater extent than single hydropriming.

## 1. Introduction

Seeds play a strategic role in plant production, agriculture, and the world’s food supply. The importance of this issue has only increased over time, particularly at the start of the 21st century, given human population growth, urbanization, and food crises. In fact, the use of improved seeds is sometimes referred to as the engine of agricultural progress because of its ability to increase productivity and stimulate economic activity in the farming world. Farmers and biologists have long sought to improve crop performance by reserving the seeds of the highest-yielding plants. In recent decades, advances in scientific research have considerably accelerated this evolution and opened new prospects for plant improvement [1,2,3]. Hence, priming seeds is a physiological method that improves plant production by modulating the metabolic activities of germination before radicle emergence [4,5], i.e., during the reversible phase of germination. During this phase, the seed can be rehydrated while retaining its ability to germinate [6]. During priming, seeds are partially hydrated to a sufficient moisture level to allow pre-germinative metabolic processes to take place but is insufficient to ensure radicle breakthrough [7]. In this field, priming, which consists of a pre-germinative treatment, is widely studied and even used to obtain better germinative performance, synchronous and homogeneous germination, and even plants that are more tolerant to abiotic stresses such as water deficit and salinity [2,7,8,9,10,11,12,13,14,15,16,17]. Seed priming methods can be divided into two groups depending on whether water uptake is uncontrolled or controlled (by PEG, for example) [15,18]. Depending on the imbibition medium, several types of priming can be distinguished: hydropriming (water), osmopriming (osmotizing agents), chemopriming, (chemical substances such as H_2_O_2_, silicon, CaCl_2_, etc.), hormopriming (phytohormones), biopriming (medium containing bacteria), or nanopriming (uses nanoparticles to assist the supply of nutrients to seeds and boost metabolic activity). Hydropriming is the most common agricultural technique, simple to apply, less expensive, and environmentally friendly. The process involves soaking seeds in water for a set period and then re-dehydrating them before sowing [4]. This type of priming does not require the use of chemicals and therefore presents no environmental or health risks. In addition to conventional hydropriming, Boucelha and Djebbar [2] employed a new treatment known as double hydropriming, which consists of performing a double cycle of seed hydration and rehydration. This priming technique significantly improved germination performance, growth, flowering, and tolerance to water stress in *Vigna unguiculata*. Osmopriming consists of subjecting seeds to an osmotic pre-germination treatment, either alone or followed by rehydration. In this case, seeds are placed in contact with osmotic agents such as polyethylene glycol (PEG), salts (KNO_3_, NaCl, KCl) or polyols (mannitol) [4,19]. The positive effects of priming are associated with various physiological, biochemical, cellular, molecular, and genetic changes. These modifications, such as reserve mobilization, endosperm degradation, membrane repair, activation of antioxidative systems, neosynthesis of certain proteins, stimulation of osmolyte synthesis, and cell cycle activation, are strongly regulated and controlled by the expression of numerous genes [10,11,12,13,14,20,21,22,23]. According to Boucelha et al. [24], the benefits of priming are also linked to changes in the redox state at the embryo level, promoting the “oxidative window”, which enables the germination process to be triggered. Because of these complex regulations and their potential consequences throughout the plant cycle, studies on molecular and genetic aspects have enabled the identification of the most relevant traits for studying the effects of seed priming. This complexity also makes it difficult to identify the links that exist between the different levels of organization and to identify an initial list of candidate genes. On the other hand, some consequences of priming may be due to DNA methylation or chromatin spatial conformation [25]. Thus, epigenetic phenomena are of key importance for understanding many phenomena in plant biology; they play a decisive role in the adaptation of plants to their environment. These epigenetic changes are modulated during development and stress exposure, resulting in a more effective defense mechanism [26,27].

However, little is known about these phenomena and the mechanisms involved during pre-germination treatment. In fact, the aim of our study was to investigate the quantitative and qualitative metabolic changes (by TLC) in the cotyledons and embryonic axes (radicle and plumule) of *Vigna unguiculata* seeds subjected to osmopriming with PEG, simple hydropriming, and double hydropriming. To our knowledge, this aspect has never been studied before.

For this work, we used the black-eyed bean *Vigna unguiculata* (L) Walp, an important staple in sub-Saharan Africa, particularly in the arid savannahs of West Africa, as our biological model. It is one of the oldest cultivated food plants and has a highly controversial origin [3,28,29]. Its seeds are a valuable source of plant proteins, carbohydrates, vitamins, minerals, and income for humans, as well as fodder for animals [30]. In many regions, young leaves, fresh or dried pods, and immature pods are also consumed [31].

## 2. Results

### 2.1. Electrolytes Leakage of Seeds

**Figure 1:**
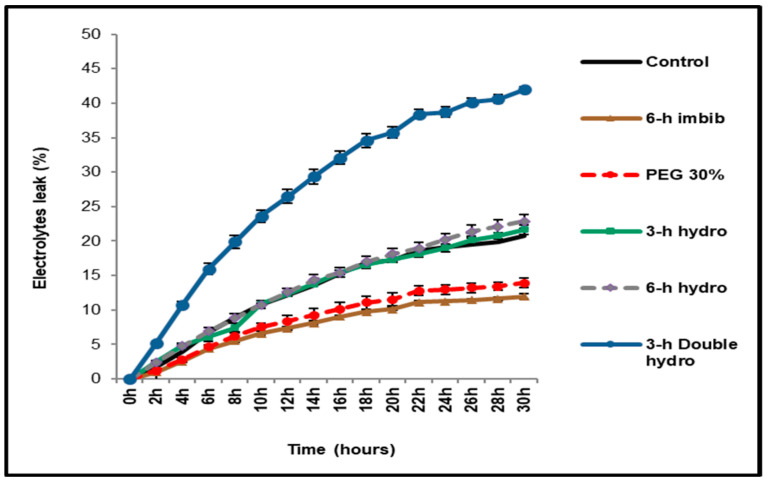
Evolution of the conductivity of the leachates from control and treated *Vigna unguiculata* seeds during germination. The error bars represent the standard error of the mean.

The leakage (or leaching) of electrolytes was measured via conductimetry. According to the curves obtained, we were able to conclude that, regardless of the type of priming (or not priming) of the black-eyed bean seeds, we observed a massive release of electrolytes in a regular and progressive manner during their imbibition. However, the rate and quantity of electrolytes released differ from treatment to treatment. Thus, this leaching was greater and faster in the double-hydroprimed seeds than in the other treatments. We also noted that the electrolytes output was lower in the control than in the primed seeds (*3 h hydro* and *6 h hydro*). Our results also indicated that seeds soaked for six hours in water or 30% PEG were characterized by low leaching of electrolytes (Figure 1).

### 2.2. Study of Water Content and Metabolites at the Embryonic Level

All measurements (weight-related and biochemical) were carried out on the embryos after seeds imbibition for two hours.

#### 2.2.1. Water Content of the Embryos

The results revealed that, compared with the radicles and cotyledons, the plumules presented the highest water content. We also observed that the cotyledons contained less water than did the radicles. On the other hand, the pre-germination treatment of *Vigna unguiculata* seeds significantly increased the water content of the cotyledons, radicles, and plumules. We noticed that, in the three organs, the sample that underwent double dehydration was characterized by the highest water content, with an increase, compared with the control, in the order of 26% in the cotyledons, 18% in the radicles, and 25% in the plumules.

**Figure 2:**
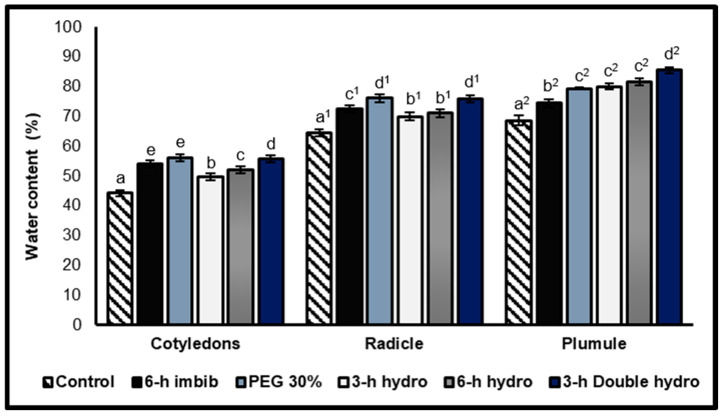
Water content of cotyledons, radicles, and plumules taken from control and treated *Vigna unguiculata* seeds after two hours of imbibition. The error bars represent the standard error of the mean. Means followed by different alphabetical letters show a significant difference between them (*p* ≤ 0.05). (**a**–**e**) Significant differences between treatments at the cotyledon level. (**a^1^**–**d^1^**) Significant difference between treatments at the radicle level. (**a^2^**–**d^2^**) Significant difference between treatments at the plumule level.

Quantitative and qualitative analyses of amino acids (including proline) and carbohydrates (soluble sugars and starch) and only quantitative analyses of proteins were carried out at the level of the cotyledons, plumules, and radicles to evaluate the possible variations induced by the different priming methods (Figure 2, Table 1).

#### 2.2.2. Quantitative Analyses of Metabolites

- Total soluble proteins contents

**Figure 3:**
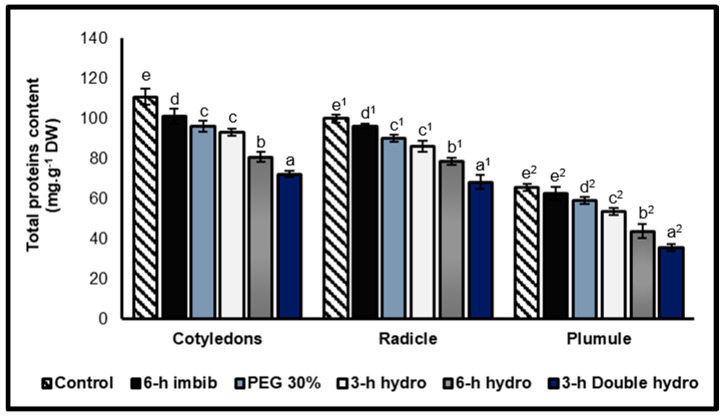
Total proteins contents of cotyledons, radicles, and plumules taken from control and treated *Vigna unguiculata* seeds. The error bars represent the standard error of the mean. Means followed by different alphabetical letters show a significant difference between them (*p* ≤ 0.05). (**a**–**e**) Significant differences between treatments at the cotyledon level. (**a^1^**–**e^1^**) Significant difference between treatments at the radicle level. (**a^2^**–**e^2^**) Significant difference between treatments at the plumule level.

The results indicated that the priming of *Vigna unguiculata* seeds strongly reduced the proteins content, for all treatments, at the level of the cotyledons and the embryonic axis (radicle and plumule), but the intensity of this reduction depended on the type of treatment. Compared with osmopriming with 30% PEG, hydropriming had a more significant effect on reducing the protein content. With respect to hydropriming, we also observed that the longer the duration of imbibition (followed by dehydration), the lower the protein content. This resulted in a decrease in total protein content, which was 18% for the cotyledons, 14% for the radicles and 16% for the plumules in *3 h hydro* seeds. This reduction was 27% for the cotyledons, 22% for the radicles and 33% for the plumules for the *6 h hydro* sample. However, a double cycle of re-dehydration was the most effective method for inducing a decrease in protein content, with rates of change of −35% for the cotyledons, −32% for the radicles and −46% for the plumules. On the other hand, our study revealed that a six-hour imbibition in 30% PEG (osmopriming) had a more significant effect on the reduction in the content of embryonic proteins (cotyledons, radicles and plumules) than a simple imbibition in distilled water (6 h imbibition), where a reduction of less than 10% was observed (Figure 3, Table 1).

- Total free amino acids contents

**Figure 4:**
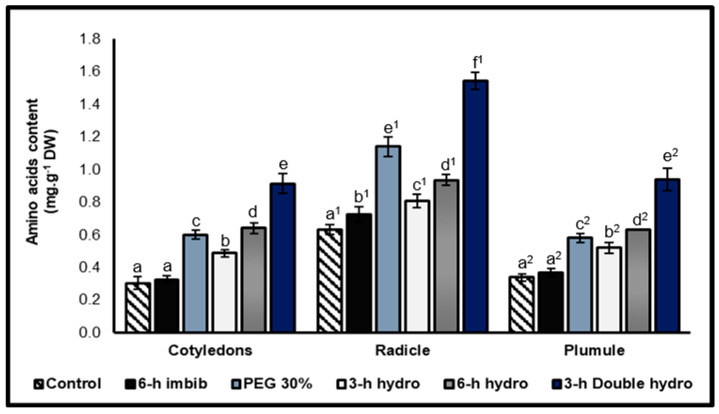
Total amino acids contents of cotyledons, radicles, and plumules taken from control and treated *Vigna unguiculata* seeds. The error bars represent the standard error of the mean. Means followed by different alphabetical letters show a significant difference between them (*p* ≤ 0.05). (**a**–**e**) Significant differences between treatments at the cotyledon level. (**a^1^**–**f^1^**) Significant difference between treatments at the radicle level. (**a^2^**–**e^2^**) Significant difference between treatments at the plumule level.

According to our results, the radicle was characterized by the highest amino acids content compared with the other organs. On the other hand, the results indicated that priming *Vigna unguiculata* seeds caused a highly significant increase (*p* < 0.001) in the amino acids content of the cotyledons, radicles and plumules. However, the magnitude of this increase differed depending on the treatment method. Compared with simple hydropriming, osmopriming had a more significant effect on the production of amino acids in the radicle. In fact, we recorded an increase of 81% in the 30% PEG treatment, whereas it was 110% in the *6 h hydro* sample and 60% in the *3 h hydro* sample. On the other hand, in the cotyledons and the plumules of the seeds that were hydroprimed after six hours of imbibition (*6 h hydro*), the amino acids content was greater than that of the samples that were osmoprimed with PEG at 30%. For simple hydropriming, we also noted that the longer the duration of imbibition preceding dehydration, the more significant the effect of priming on the accumulation of amino acids. Very strong stimulation of amino acids production was observed in the cotyledons and embryonic axes of double-hydroprimed seeds, with a rate of variation of +200% in the cotyledons compared with the control, +145% in the radicles and +177% in the plumules. Note that a simple six-hour imbibition in distilled water induced a very slight increase in the amino acids content (+15% in the radicles) (Figure 4, Table 1).

- Total amino acids: total proteins ratio

**Figure 5:**
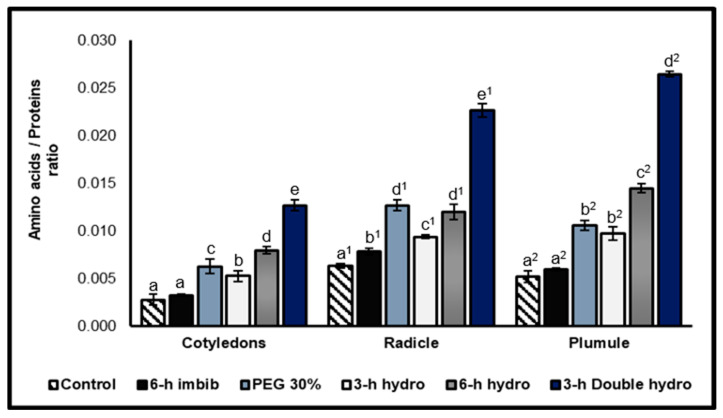
Total amino acids/total proteins ratio. The error bars represent the standard error of the mean. Means followed by different alphabetical letters show a significant difference between them (*p* ≤ 0.05). (**a**–**e**) Significant differences between treatments at the cotyledon level. (**a^1^**–**e^1^**) Significant difference between treatments at the radicle level. (**a^2^**–**d^2^**) Significant difference between treatments at the plumule level.

The amino acids/total proteins ratio made it possible to weigh the accumulation of amino acids in relation to the quantity of proteins. The values of these ratios clearly revealed the anticorrelation between the amino acids content and total proteins content. Our results indicated that priming and, more particularly, double hydropriming significantly increased the value of the ratio, with a rate of variation of +352% in the cotyledons, +259% in the radicles, and +408% in the plumules compared with the control (Figure 5, Table 1).

- Free proline contents

**Figure 6:**
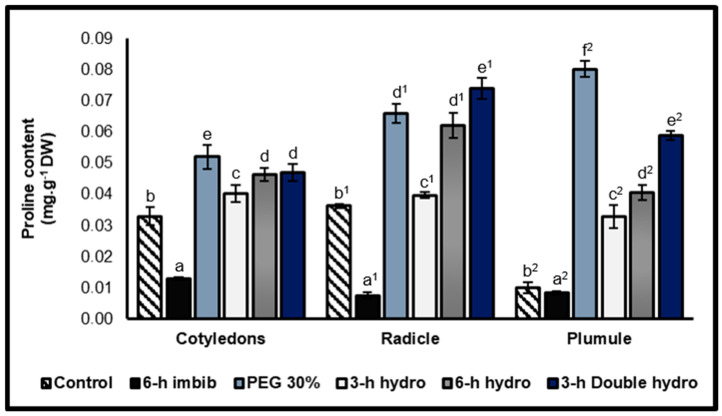
Free proline contents of cotyledons, radicles, and plumules taken from control and treated *Vigna unguiculata* seeds. The error bars represent the standard error of the mean. Means followed by different alphabetical letters show a significant difference between them (*p* ≤ 0.05). (**a**–**e**) Significant differences between treatments at the cotyledon level. (**a^1^**–**e^1^**) Significant difference between treatments at the radicle level. (**a^2^**–**f^2^**) Significant difference between treatments at the plumule level.

Our results revealed that proline was more abundant in the embryonic axis (radicle and plumule) than in the cotyledons. Second, the results and statistical analyses indicated that priming significantly induced the biosynthesis and accumulation of free proline. However, the increase in free proline content differed depending on the organ and priming method. Notably, in the radicles, double dehydration induced the greatest increase in the free proline content compared with that of the control, with a rate of variation of +105%. Therefore, in the cotyledons, the plumules were the 30% PEG sample, which was characterized by the highest content of free proline, with increases in the cotyledons and 58% and 702% in the plumules. With respect to simple hydropriming, we observed that imbibition for six hours followed by dehydration (*6 h hydro*) was more effective than imbibition for three hours (*3 h hydro*) in inducing the accumulation of free proline in the three organs. This was confirmed by the increase in its content, which was 41% (cotyledons), 72% (radicles), and 306% (plumules) in the *6 h hydro* sample, whereas it was 22% (cotyledons), 10% (radicles), and 228% (plumules) in the *3 h hydro* sample. Compared with the control, simple six-hour imbibition in distilled water caused a significant decrease in the free proline content: −61% in the cotyledons, −79% in the radicles, and −17% in the plumules (Figure 6, Table 1).

- Free proline: total amino acids ratio

**Figure 7:**
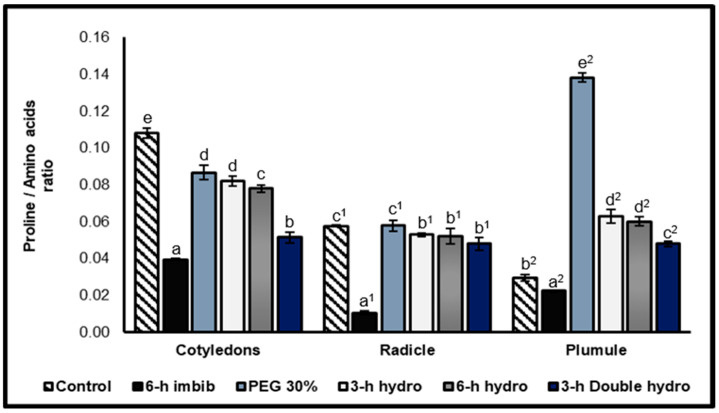
Free proline/total amino acids ratio. The error bars represent the standard error of the mean. Means followed by different alphabetical letters show a significant difference between them (*p* ≤ 0.05). (**a**–**e**) Significant differences between treatments at the cotyledon level (**a^1^**–**c^1^**) Significant difference between treatments at the radicle level/ (**a^2^**–**e^2^**) Significant difference between treatments at the plumule level.

The proline/total amino acids ratio allows for the weighing of the accumulation of proline in relation to the quantity of total amino acids. This study thus investigated whether proline accumulates in the same way as other amino acids or whether its accumulation is independent of that of total amino acids. The highly variable values of this ratio clearly revealed that the increase in the content of free proline was independent of that of total amino acids. Our results revealed that the ratio decreased in the cotyledons and radicles of treated seeds compared with those of the controls. This reduction was greatest when the seeds were soaked for 6 h (−64% for the cotyledons and −82% for the radicles). On the other hand, in the plumules, this ratio increased in the samples that had undergone priming and particularly in the plumules of seeds that were osmoprimed with PEG at 30% (+307%) (Figure 7, Table 1).

- Starch contents

**Figure 8:**
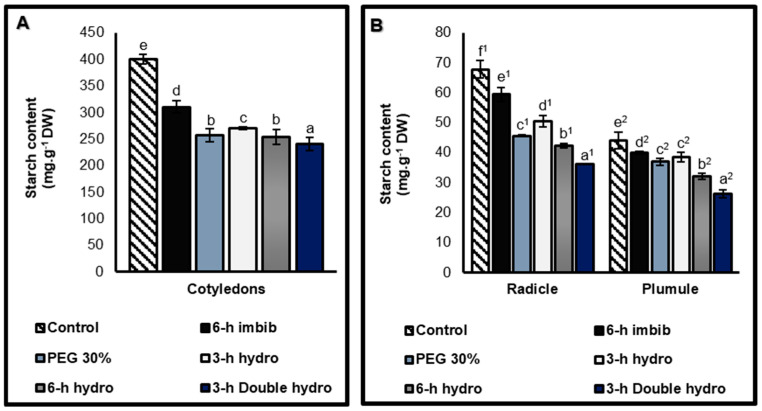
Starch contents of cotyledons (**A**), and radicles and plumules (**B**) taken from control and treated *Vigna unguiculata* seeds. The error bars represent the standard error of the mean. Means followed by different alphabetical letters show a significant difference between them (*p* ≤ 0.05). (**a**–**e**) Significant differences between treatments at the cotyledon level. (**a^1^**–**f^1^**) Significant difference between treatments at the radicle level. (**a^2^**–**e^2^**) Significant difference between treatments at the plumule level.

The cotyledons were richer in starch than the radicles and plumules were. Furthermore, the results and statistical analyses indicated that the priming of *Vigna unguiculata* seeds caused a decrease in the starch content in all three organs. However, the importance of this reduction depended on the type of treatment. Thus, hydropriming (*6 h hydro*) had a more significant effect on the reduction in the quantity of starch than osmopriming with PEG did at 30%, and more particularly in the plumules, the reduction was 16% in the PEG 30% sample compared with 27% in the *6 h hydro* samples. With respect to hydropriming, we also observed that the longer the duration of imbibition, the more significant the reduction in starch content. This was confirmed by the reduction in starch content, which was 32% (cotyledons), 26% (radicles), and 12% (plumules) in seeds soaked for three hours, whereas it was 37% (cotyledons), 38% (radicles), and 27% (plumules) when the imbibition time was six hours. However, the double cycle of dehydration most significantly affected the reduction in starch, with 40% in the cotyledons, 47% in the radicles, and 40% in the plumules. Compared with treated samples, simple six-hour imbibition in distilled water (6 h imbib) did not cause a significant reduction in starch content (Figure 8, Table 1).

- Total soluble sugars contents

**Figure 9:**
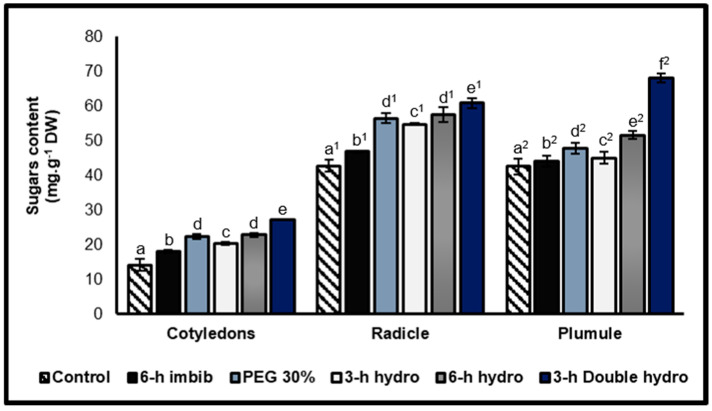
Soluble sugars contents of cotyledons, radicles, and plumules taken from control and treated *Vigna unguiculata* seeds. The error bars represent the standard error of the mean. Means followed by different alphabetical letters show a significant difference between them (*p* ≤ 0.05). (**a**–**e**) Significant differences between treatments at the cotyledon level. (**a^1^**–**e^1^**) Significant difference between treatments at the radicle level. (**a^2^**–**f^2^**) Significant difference between treatments at the plumule level.

Firstly, we observed that the embryonic axis (especially the radicle) is richer in soluble sugars than cotyledons. Secondly, the results and statistical analyses indicated that any type of pre-germination treatment of black-eyed bean seeds significantly stimulated the biosynthesis and accumulation of soluble sugars in the cotyledons, radicles, and plumules. The magnitude of this increase varied depending on the method of treatment applied. Our results indicated that the greater the duration of imbibition before dehydration (simple hydropriming) or the greater the number of hydration—dehydration cycles (double hydropriming) was, the more significant the effect of seeds priming. Compared with those of the other treatments, the cotyledons, radicles, and plumules of the double-hydroprimed seeds presented the highest contents, with variation rates reaching +91% in the cotyledons, +42% in the radicles, and +60.2% in the plumules compared with those of the control. However, a simple six-hour soaking in distilled water induced a very slight increase in the soluble sugars content: +27% in the cotyledons, +9% in the radicles, and +4% in the plumules (Figure 9, Table 1).

- Soluble sugars: starch ratio

**Figure 10:**
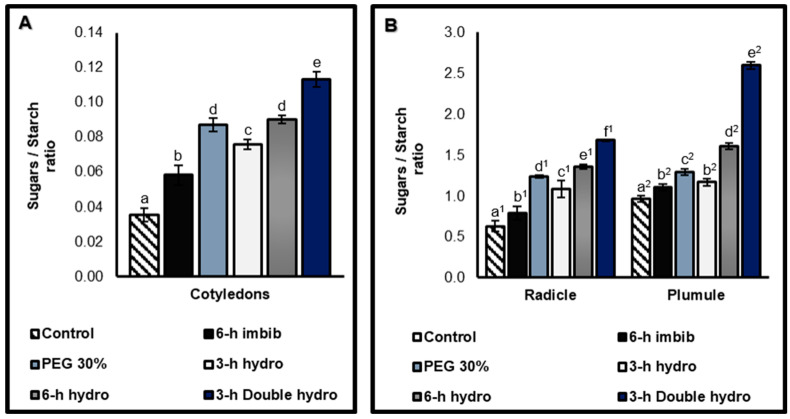
Soluble sugars/starch ratio of cotyledons (**A**), and radicles and plumules (**B**) taken from control and treated *Vigna unguiculata* seeds. The error bars represent the standard error of the mean. Means followed by different alphabetical letters show a significant difference between them (*p* ≤ 0.05). (**a**–**e**) Significant differences between treatments at the cotyledon level. (**a^1^**–**f^1^**) Significant difference between treatments at the radicle level. (**a^2^**–**e^2^**) Significant difference between treatments at the plumule level.

This ratio allows for the weighing of the accumulation of soluble sugars in relation to the quantity of starch (Figure 10). The results indicated that there is a correlation between the content of soluble sugars and that of starch. Compared with the control, priming and, more particularly, double dehydration significantly increased the value of the ratio, with rates of variation of +223% in the cotyledons, +166% in the radicles, and +169% in the plumules (Figure 10, Table 1).

#### 2.2.3. Qualitative Analyses of Metabolites

- Amino acids composition

We separated and identified by thin-layer chromatography (TLC) the amino acids contained in the cotyledons, radicles, and plumules of the control and treated seeds (Table 2).

*-Cotyledons:* Four amino acids, proline, cysteine, ornithine, and glycine, were present in the cotyledons regardless of the treatment. The ornithine and glycine appeared to be more abundant in the cotyledons of seeds that had undergone double hydropriming. A fifth amino acid, threonine, appeared following the *6 h hydro* or *3 h double hydro* samples. Valine was present only in the cotyledons of seeds that had undergone double hydropriming (Table 2).

*-Radicles:* They contained eight amino acids, namely, histidine, aspartate, threonine, alanine, valine, methionine, tryptophan, and proline, regardless of the type of treatment. Isoleucine appeared as a ninth amino acid in the radicles of osmoprimed or hydroprimed seeds. However, the majority of these amino acids, particularly isoleucine, appeared to be more abundant in the radicles of seeds that had undergone double rehydration than in those of the other primed seeds. Double dehydration also induced the appearance of another amino acid, cysteine, in the radicles. Similarly to the results for the cotyledons and the plumules, we observed that imbibing the seeds for six hours in distilled water (6 h imbib) did not cause any qualitative modification of the amino acids composition in the radicles (Table 2).

*-Plumules:* In all samples (control and treated), we noted the presence of arginine, threonine, valine, and proline. These amino acids were also more abundant in the plumules of seeds that had undergone double hydropriming. On the other hand, double dehydration allowed the appearance of cysteine, serine, and tryptophan in the plumules (Table 2).

- Composition of soluble sugars

The soluble sugars of the different organs of the embryo were separated and identified via thin-layer chromatography (Table 3).

*-Cotyledons:* In all samples (control and treated), we noted the presence of galactose and a derivative of glucose (by oxidation), gluconic acid. Compared with that of the cotyledons of the control seeds, the simple sugars composition of the cotyledons was modified when the seeds underwent priming except for the *3 h hydro* treatment. The composition of the seeds soaked for 6 h presented the same profile as the control. Following osmopriming, the composition was characterized by the disappearance of maltose and only had two oses (or derivatives): galactose and gluconic acid. Dehydration after 6 h of imbibition (*6 h hydro*) induced the formation of sucrose, but double hydropriming modified the sugars composition of the cotyledons the most, since we observed the appearance of sucrose, fructose, and ribose (Table 3).

*-Radicles:* Like amino acids, thin-layer chromatography (TLC) has shown that the radicle is the organ whose composition of soluble sugars varied the most. Regardless of the treatment (or not), the radicles contained three oses (maltose, galactose, and fructose) and a glucose derivative (gluconic acid). Only double hydropriming caused the appearance of two new oses, glucose and xylose (Table 3).

*-Plumules:* The plumules of the control seeds contained only one ose, glucose. If the seeds were soaked for 6 h or underwent osmporiming or simple hydropriming for 3 h, the presence of gluconic acid and maltose was noted. A 6 h period of hydropriming induced the appearance of trehalose. This composition was further enriched following double hydropriming since we revealed, in addition to glucose, maltose, gluconic acid, and trehalose, the presence of three new oses: arabinose, rhamnose, and ribose (Table 3).

**Note:** It is very likely that the number of amino acids and sugars present in different organs is relatively high, which cannot be demonstrated by simple one-dimensional TLC.

## 3. Discussion

Our biochemical analysis of the cotyledons, radicles, and plumules of control and treated seeds aimed to understand the biochemical mechanisms governing the priming of black-eyed bean seeds, *Vigna unguiculata*, to explain the beneficial effects of priming on germination, development, growth, and seed vigor index under favorable and stressful conditions, demonstrated through our previous work under the same conditions [2,24,32]. Thus, we have shown that double hydropriming was more effective in improving these parameters, compared to the control sample. In this context and under favorable conditions, a variation rate of +28% was recorded for germination capacity, +55% for radicle length, +101% for radicle weight growth and +98% for the seeds vigor index.

### 3.1. Effect of Priming on Electrolytes Release

Conductometric measurements have already shown that during germination, seeds lose nutrients in the imbibition medium [33]. Most of the soluble solutes that escape from seeds are sugars and amino acids, in addition to most of the mineral ions present in the seed, such as Ca^2+^, Na^+^, and especially K^+^, whose essential role is known during germination [34]. The imbibition of the grains leads to rapid rehydration of the cell membranes, which are still disorganized at the beginning, promoting the passive exit of mineral elements and other solutes [35]. Nutrients release may be due to tubular channels present in the membrane phospholipid bilayer, which open under normal conditions (imbibition) and under stressful conditions such as dehydration [36].

Paradoxically, our study shows that the leaching of electrolytes is positively correlated with the speed of germination. We thus observe that the hydroprimed seeds, which are characterized by the best germination performance, present a strong output of electrolytes. This is particularly remarkable for double-hydroprimed seeds, in which leaching is intense. Therefore, as part of simple hydropriming (*3 h hydro* and *6 h hydro*), the abundance of these electrolytes in the imbibition medium is positively correlated with the duration of seed hydration.

Seed priming induces the activation of pre-germinative processes, such as the activation of enzymes involved in the degradation and mobilization of reserves [22,32,37]. Consequently, compared with unprimed seeds, treated seeds are rich in degradation products (sugars, amino acids, etc.), which could explain the rapid leaching of nutrients in seeds that have undergone treatment. Furthermore, we have shown in previous experiments that priming seeds undergo a physical disorganization of their seed coat structure [38] leading to a loss of adherence, unrelated to the alteration in membrane integrity. This observation may explain a greater leakage of electrolytes without affecting germination performance.

### 3.2. Effect of Priming on Water Absorption

Our results indicated that the water content was greater in the cotyledons, radicles, and plumules of seeds than in those of control seeds. These observations could be explained by the results of the imbibition kinetics of *Vigna unguiculata* seeds obtained by Boucelha and Djebbar [2], who reported that water absorption is much faster in primed seeds than in control seeds. Our results agree with the results of Gelormini [39], who suggested that seed priming accelerated water uptake during imbibition. This rapid water uptake could be due to the high permeability of the seed coats of primed seeds compared with non-primed seeds (controls).

Physiologically, these results indicate that during imbibition, the embryo absorbs water more quickly than cotyledons do. This high-water content of the embryo allows good cellular turgor, which is necessary for the expansion and emergence of the radicle. This could also explain the positive effects of priming on the emergence and vigor of seeds [2,24].

### 3.3. Effect of Priming on the Reserve Hydrolysis

Our study led to the conclusion that seed priming, particularly double hydropriming, stimulated the hydrolysis of cotyledonary and embryonic proteins and carbohydrate reserves. At the physiological level, previous studies have clearly shown that priming results in strong synthesis and activation of enzymes involved in the degradation and mobilization of proteins (proteases), carbohydrate (alpha-amylase), and lipid (lipase) reserves. The products of this hydrolysis (amino acids, soluble sugars, and fatty acids) are used during germination [3,10,22,40,41,42,43,44].

Bradford [4] and Hanson [45] suggested that the activation of alpha-amylase during the first imbibition preceding dehydration corresponds to the vigor set during hydropriming. Thus, an increase in α-amylase activity in primed seeds has been reported by numerous authors [9,41,42,44]. This enzyme allows the hydrolysis of starch, which leads to an increase in the soluble sugars content. These sugars are involved in the metabolic processes involved in the growth of the embryo. They are considered the main source of energy; in fact, this accumulation would partly explain the increase in the speed of germination of primed seeds observed by [2,17,42].

On the other hand, compared with that of amylases, the effect of priming on the activity of proteases has rarely been studied. Some authors have shown that seed priming promotes protein hydrolysis [22,37,43,46]. During germination, protein hydrolysis releases a broad spectrum of amino acids that ensure the transport of nitrogen from storage organs to growing organs. These amino acids, subject to various interconversions, are mobilized for the synthesis of new proteins and key metabolites [34,47]. On the other hand, a proteomic analysis of primed seeds during germination revealed an increase in the degradation products of the β-12S subunit of cruciferin in *Arabidopsis thaliana* and of the β-11S subunit of globulin in sugar beet [22]. These findings suggest that the enzymes involved in the mobilization of reserve proteins are either synthesized or activated at the cotyledon level during seed priming.

For our study, the hydrolysis of proteins and carbohydrates (starch) were translated into a high content of amino acids and soluble sugars at the level of the three organs of the embryo, particularly at the level of the radicle. This accumulation is accentuated in the case of double re-dehydration. The availability of these hydrolysates can explain the improvement in the germination performance of primed seeds, as observed through our own results [2,24,37]. This accumulation also contributes to osmotic adjustment under stressful conditions [39,44]. On the other hand, regarding the radicle and plumule, the increase in amino acids and sugars contents may also be due to neosynthesis.

However, our study revealed that a double cycle of re-dehydration had a greater effect on the hydrolysis of reserves than did simple hydropriming (a single cycle of re-dehydration), which also confirms that the effectiveness of priming depends on the number of dehydration cycles and not imbibition duration.

The ratios of total amino acids/total proteins and soluble sugars/starch confirmed that the embryos of the primed seeds were characterized by high contents of amino acids and soluble sugars compared with those of the controls. This accumulation is linked to the contents of total proteins and starch, of which the priming of the seeds favors their hydrolysis at the embryonic level. On the other hand, we noticed that amino acids and soluble sugars were highly abundant in the radicle and more particularly in the double-hydroprimed treatment. This confirmed the effectiveness of priming on the mobilization of reserve degradation products towards the embryonic axes.

### 3.4. Effect of Priming on Free Proline Accumulation

Proline accumulation is a nearly universal response of plants to a wide range of stresses, including water stress. This amino acid plays several roles in the dehydration of the cellular environment. In addition to its involvement in osmoadjustment for the maintenance of cellular turgor, proline plays an important role in osmoprotection [48]. Our results revealed that priming, particularly osmopriming with PEG at 30% and double dehydration, promoted the production of free proline in the cotyledons, radicles, and plumules. Seed priming results in the stimulation of the biosynthesis of osmolytes such as free proline to ensure osmotic adjustment under stressful conditions [21,39,49]. The free proline/total amino acids ratios, which were lower than those of the control, showed that, in the case of the cotyledons and the plumules, the accumulation of proline was linked to an overall increase in total amino acids following hydrolysis proteins. In the case of the plumules, this ratio was greater and reflected a neosynthesis of proline for all the treatments, apart from an imbibition of 6 hours. In this organ, de novo synthesis is particularly important when the embryo undergoes osmotic stress during treatment with 30% PEG. The stimulation of free proline biosynthesis in primed seeds is correlated with high expression of two genes and with high levels of mRNAs corresponding to enzyme activity [39]. Thus, Kubula et al. [50] explained the increase in proline content in primed seeds by strong regulation of the expression of the P5CSA gene encoding pyrroline-5-carboxylate (P5C) synthase A, a key enzyme of proline biosynthesis, as well as the repression of the PDH gene.

### 3.5. Effects of Priming on Amino Acids Composition

The qualitative analysis of amino acids by TLC revealed that priming, particularly double dehydration, caused considerable modifications in the amino acids composition by favoring the appearance of certain amino acids. In cotyledons, double hydropriming induced the appearance of threonine and valine as well as the overproduction of ornithine and glycine. These are essential amino acids whose role is fundamental and varies in the seed. Threonine is a precursor of glycine and serine and plays a role in the defense against biotic and abiotic stresses [51,52]. This amino acid is metabolized mainly in plastids; hence, it is present in cotyledons [53]. Valine affects the speed of root formation and the growth rate of plants and plays a role in tissue repair and energy production [54]. Ornithine is a non-proteinogenic amino acid and a proline precursor that plays a fundamental role in osmotic adjustment, membrane stabilization, proteins conformation, and detoxification in plants [55,56]. Glycine plays a crucial role in the formation of plant tissues. It contributes to the vegetative growth of plants and has a significant chelating effect. It also intervenes in resistance systems facing unfavorable conditions, playing an osmoprotective role [57,58]. These amino acids produced in cotyledons are transported, during germination, to the embryonic axes to fulfill their roles. In the radicle, any type of priming promotes the production of isoleucine, and double hydropriming induces the appearance of cysteine as well as the overproduction of other amino acids, such as valine, threonine, and methionine. Isoleucine is involved in vegetative growth from the start of vegetative growth until harvest and is used for energy production [59,60]. Cysteine is a very abundant amino acid in plants; it reduces sulfur in plants and participates in the assimilatory reduction of sulfate in chloroplasts [61,62,63]. Cysteine derives all the other sulfur compounds of the cell, particularly methionine and glutathione; thus, it represents a metabolic precursor of different essential biomolecules, such as vitamins, cofactors, antioxidants, and numerous defense compounds [63,64,65]. It also plays a role in DNA repair, fatty acid synthesis, and cellular detoxification [63]. In plumules, double dehydration is the only treatment that induces the production of certain amino acids, such as cysteine, serine, and tryptophan, as well as the overproduction of arginine, valine, and threonine. Serine plays a fundamental role in development, metabolism, and signaling in living organisms. It stimulates photosynthesis and plant tolerance to biotic and abiotic stresses. It plays an important role in the hormonal balance of plants and has an effect on the reduction of sulfur [66,67]. It is a precursor to cysteine. Tryptophan is the main precursor of indole acetic acid, the most important plant-growth substance of the auxin group. This hormone is synthesized in stems and young leaves, as well as in developing seeds [68,69]. Tryptophan is also involved in plant resistance to biotic and abiotic stresses [70]. These results revealed that there was a relationship between seed priming and amino acids composition. Indeed, the production of valine, glycine, isoleucine, and tryptophan could help accelerate germination and stimulate growth by promoting the biosynthesis of growth hormones such as auxins. On the other hand, Boucelha and Djebbar [2] showed that priming, particularly double hydropriming, allows the tolerance of primed seeds to osmotic stress (PEG at 20%), which could be due to the amino acids involved in resistance to unfavorable conditions, such as ornithine, serine, tryptophan, glycine, cysteine, and threonine.

### 3.6. Effects of Priming on Soluble Sugars Composition

Carbohydrates are essential for the development of the embryo since they are the main source of energy and represent, on the one hand, elements of support and structure and, on the other hand, molecules of protection and cellular recognition [71]. Our study revealed that hydropriming and, above all, double hydropriming caused clear qualitative modifications in soluble sugars in the cotyledons, plumules, and radicles. In control seeds, in addition to gluconic acid, maltose, a product of starch hydrolysis, is found in organs rich in this reserve polysaccharide (cotyledons and radicles). The breakdown of maltose provides for glucose molecules to synthesize other sugars necessary for germination and embryo development. At the plumule level, we found only glucose. In non-green organs, glucose comes from carbohydrate stores and is involved in cell growth and the maintenance of energy and metabolic homeostasis [72]. Thus, we can assume that at the level of the cotyledon and the radicle, the glucose has undergone aldehydic oxidation to give gluconic acid, which is not the case for the plumule. Gluconic acid was detected in all organs of treated or untreated seeds, except in the plumules of control seeds. This glucose derivative is very common in plants. It plays a key role in the nucleotide sugars biosynthesis pathway and is the common precursor for arabinose, xylose, galacturonic acid, and apiose residues found in the cell wall. It is considered a detoxification agent under conditions of oxidative stress and a regulator of the development and biosynthesis of polysaccharides, as well as inter- and intra-cellular signaling [73]. In addition to the plumule, galactose is present regardless of the treatment (or not). This ose, which is incorporated into certain oligosaccharides, is present at the cell wall [74]. Maltose was found, overall, in all organs and regardless of the type of treatment. However, its presence is much more important in the plumule, particularly if the seed has undergone a double-hydropriming treatment. This finding reflected strong hydrolysis by the activation of amylases and was confirmed by our quantitative study of starch and soluble sugars. Fructose appeared only at the radicle level, regardless of the type of treatment (or not). This sugar is a natural ketone sugar and is part of the composition of sucrose. It is involved in the preservation of homeostasis in plants under stressful conditions [75]. Interestingly, glucose was present in large quantities at the level of the radicle of double-hydroprimed seeds. This abundance could be due to a high demand following the increase in respiratory activity, as well as the acceleration of the hydrolysis of carbohydrate reserves during priming. Sucrose only appeared in the cotyledons of seeds that had undergone *6 h hydro* or *3 h double hydro*. Sucrose is the carbon molecule most frequently found in plants. It plays a central role in plant growth and development [76]. Sucrose is used as a storage disaccharide for glucose and cellulose as well as a building block of plant cell walls. It also represents a form of reserve in stems and roots. Given that this sugar functions as a primary transport sugar [77], we can hypothesize that hydropriming favors the transport of carbohydrates towards the sink organs, which are the plumule and the embryo. Trehalose also appeared only if the seeds underwent 6 h simple hydropriming or double hydropriming but only in the plumules. This ose plays a central role in the osmoprotection of plants under stressful conditions. It is known for its accumulation in drought-adapted plants. It forms a gel phase when cells dehydrate, which prevents rupture of the organelles in the cell [78]. Other simple sugars only appeared following double hydropriming, which gives them interest as discriminating molecules. These are ribose, xylose, arabinose, and rhamnose, with significant relative abundances. Ribose is present in cotyledons and plumules. This ose is part of the composition of ribonucleic and deoxyribonucleic acid RNA and DNA. It is also a component of ATP (adenosine triphosphate), NADH, and various other molecules important in metabolic processes [79]. The appearance of ribose in treated seeds could explain the acceleration of DNA replication and the increase in RNA synthesis already shown by De Castro et al. [20] and Black and Bawley [7]. This could also explain the improved root growth of seedlings from primed seeds. Thus, this abundance of ribose and glucose in the primed seeds could confirm the acceleration of respiratory activity, which is accompanied by a marked increase in the quantity of ATP in the embryonic tissues during priming, as previously demonstrated by Corbineau et al. [80]. Xylose, which is found only at the level of the radicle, is one of the constituents of hemicelluloses in cell walls [81]. Arabinose and rhamnose, observed only at the level of the plumule, are constituents of the external skeleton of plant cells, pectins, and hemicellulose of cell walls [81,82]. They also play important roles in cell aggregation [83].

## 4. Materials and Methods

The present study focused on seeds of the black-eyed bean *Vigna unguiculata* (L.) Walp, variety Lojy Bemaso, native to Madagascar. Healthy seeds of the same size were selected and rinsed with bleach to decontaminate them.

### 4.1. Seed Priming

**Figure 11:**
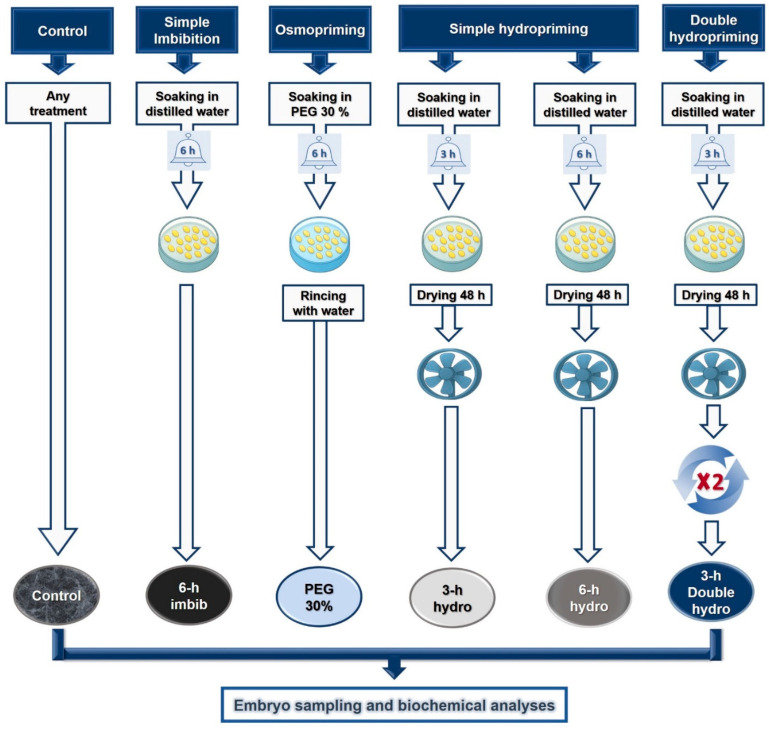
Graphical abstract explaining the protocol used to apply different types of pre-germination treatments to *Vigna unguiculata* seeds.

The concentration of polyethylene glycol (PEG) and the duration of imbibition were determined following several preliminary trials [2]. For priming efficiency, seeds were imbibed in germination trays (20 cm × 20 cm) lined with a triple layer of absorbent paper and covered with glass plates. Soaking was performed by adding 100 mL of distilled water or PEG_6000_ solution. For all types of treatment applied, imbibition took place at 26 °C in a laboratory oven (Model UFE 500, Memmert GmbH, Schwabach, Germany) (Figure 11).

#### 4.1.1. Osmopriming

The treatment of seeds involved imbibition for 6 h (reversible germination phase) in a 30% PEG_6000_ solution (PEG 30%) corresponding to −1.027 MPa.

#### 4.1.2. Hydropriming

Two samples of seeds underwent simple rehydration, which involved soaking the seeds in distilled water for 3 h (*3 h hydro*) or 6 h (*6 h hydro*). This imbibition is followed by drying under ventilation until the seeds regain their initial weight.

#### 4.1.3. Double Hydropriming

Another sample of seeds was subjected to double hydropriming, i.e., the seeds were soaked in distilled water for 3 h and then dehydrated, and the operation was repeated a second time (*3 h double hydro*).

We considered two control samples: the first (*control*) had undergone no treatment prior to the germination of the seeds, and the second (*6 h imbib*) for which the seeds were soaked in water for 6 h prior to germination.

### 4.2. Electrolytes Leakage from Seeds

This allowed us to study the physiological state of the seeds and was able to provide information concerning the vigor of the seeds and the progress of germination, which is manifested by the release of electrolytes, which increases over time. This measurement reflects the loss of electrolytes by these seeds soaked in deionized water. The leakage of electrolytes from the seeds to the external environment was measured following the protocol described by Dionisio-Sese and Tobita [84]. First, after being washed with distilled water, 20 seeds from each treatment were soaked in 20 mL of distilled water in tubes at room temperature. The electrical conductivity of the soaking water (ECi) was measured every two hours for 36 h via a conductivity meter (Model Extech EC500, Extech Instruments, Taipei, Taiwan), and the values are expressed in microSiemens per centimeter (μS/cm). The seeds are then boiled at 100 °C for two hours until all the cellular structures are destroyed. After cooling, the total conductivity (ECt) was also measured.

The leakage of electrolytes is determined by the ratio between the conductivity of the seeds placed in unboiled water (ECi) and that of the seeds put to boil (ECt) according to the following equation:

Electrolyte leakage (%) = ECi × 100/ECt

### 4.3. Sampling of Embryos

The control and primed seeds were soaked in distilled water for two hours in beakers, after which the embryos (free of cotyledons) were removed via forceps. This operation was carried out under low-temperature conditions (in crushed ice) to prevent the absorption of water by the embryos as well as their development so that all the seeds are at the same physiological stage.

Our biochemical analyses were carried out on three organs separately: the radicle (future root), the plumule (future leaves) and the cotyledons (reserve organs).

### 4.4. Quantitative Analyses

#### 4.4.1. Water Content of the Embryos

After the average weights of the fresh plant material (FW) and dry plant material (DW) of the cotyledons, radicles, and plumules (separately) were obtained from one hundred seeds from each treatment, after two hours of imbibition, the water content of each organ was calculated according to the following relationship:

Water content (%) = (FW − DW/FW) × 100

#### 4.4.2. Determination of Soluble Proteins

Total proteins content was determined according to the Bradford method [85]. One hundred milligrams of radicles, 50 mg of plumules, or 100 mg of cotyledons were ground separately and cooled in Tris-HCl buffer (pH 8.1). Centrifugation was then carried out at 12,000 rpm for 20 min at 4 °C. The supernatant, which contained the total proteins to be measured, was recovered. A total of 10 µL of proteins extract was added to 3 mL of Bradford reagent. The blue color developed immediately, and after incubation for 5 to 60 min, the absorbance was read on an absorption spectrophotometer at 595 nm (Model 6305, Jenway, Staffordshire, UK).

#### 4.4.3. Determination of Total Amino Acids

The technique used was that developed by Yemm and Cocking [86], modified by Rosen [87]. For each treatment, 150 mg of radicles, 60 mg of plumules, and 150 mg of cotyledons were ground (separately) in 2 mL of distilled water for the radicles and cotyledons and in 1 mL for the plumules. The tubes were then placed in a boiling water bath (Model WNB14_B02643, Memmert GmbH + Co. KG, Schwabach, Germany) at 100 °C for one hour to destroy the cellular structures. After cooling, centrifugation was carried out. The first supernatant was collected, and the pellet was ground a second time, in 2 mL of 90% ethanol for the radicles and cotyledons and in 1 mL for the plumules. The tubes were then centrifuged after a second water bath for 15 min at 60 °C. The second supernatant was added to the previous one to be used for the actual assay. An aliquot of the extract (400 μL) was mixed with 500 μL of citrate buffer (160 mM, pH 4.6). After homogenization, 1 mL of the ninhydrin-ascorbic acid reaction mixture was added to the ground material. Then, the tubes were shaken and placed in a boiling water bath for 20 min. After cooling, 3 mL of 70% ethanol was added to the radicle and cotyledon extracts, and only 1 mL was added to the plumule extracts. The absorbance of the purple chromogen was measured at 570 nm.

#### 4.4.4. Determination of Free Proline

The method used was that of Bates [88], modified by Magné and Larher [89], in such a way as to avoid interference with sugars. The same amino acids extracts were used for the proline assay. Five hundred microliters of proline extract were added to 1 mL of ninhydrin reagent. The tubes were homogenized and placed in a water bath at 95 °C for 20 min. Then, 3 mL of toluene was added after vortexing, and two phases developed. The upper organic phase containing proline was removed, while the lower aqueous phase was eliminated. The optical density of the samples was determined via spectrophotometry at a wavelength of 520 nm.

#### 4.4.5. Determination of Total Soluble Sugars

The determination of carbohydrate content was performed via colorimetry according to the anthrone method of McCready [90]. The same amino acids extracts were used for the determination of soluble sugars. To 1 mL of carbohydrate extract (diluted 1/100), 2 mL of anthrone reagent (0.2 g in 100 mL of 95% sulfuric acid) was added. The tubes were then placed in a water bath at 100 °C for 10 min to allow the development and stabilization of the color. After cooling, the optical densities were read with a spectrophotometer at 620 nm.

#### 4.4.6. Determination of Starch

After extraction of the soluble sugars, the starch contained in the pellet underwent gentle acid hydrolysis, and the number of glucose units released was measured as previously described. The dosages used were based on the reducing properties of the sugars. For starch hydrolysis, 2 mL of 35% perchloric acid (for the radicles and cotyledons) and 1 mL for the plumules were added to the pellets (from the extraction of soluble sugars). The homogenates were then stirred for 15 min in crushed ice. After cooled centrifugation, the supernatant was collected and stored. The operation was repeated by adding the same volume of perchloric acid to the pellets, and after hydrolysis for 15 min, centrifugation was carried out, and the second supernatant was added to the previous one. The dosage is carried out in the same way as in the case of soluble sugars.

### 4.5. Quantitative Analyses

The identification of soluble sugars and amino acids in the radicle, plumule and cotyledon of each treatment was carried out via thin-layer chromatography (TLC).

#### 4.5.1. Identification of Amino Acids by TLC

The silica plates were immersed in an eluting solvent containing butanol, acetic acid, and distilled water (70/18/12, *v*/*v*/*v*). The development was carried out by spraying the amino-acid-revealing reagent containing 1% ninhydrin in acetone, and the spots appeared purple after passing through an oven at 120 °C.

#### 4.5.2. Identification of Soluble Sugars via TLC

The mobile phase consisted of chloroform, acetic acid, and distilled water (3/3.5/0.5, *v*/*v*/*v*). The identification of soluble sugars was carried out using thymol prepared in alcohol and sulfuric acid (0.5 g of thymol, 95 mL of 96% ethanol, and 5 mL of pure sulfuric acid). After the plates were placed in the oven at 80 °C for 15 min, the spots appeared pink.

### 4.6. Statistical Test

The experiments were repeated at least five times. The results of these manipulations were presented in the form of curves or histograms representing the average values. Bars represent standard errors. One-way analysis of variance (ANOVA) and Student’s t-test was used to assess differences in the different parameters between control and each treatment or between two treatments. All statistical analyses were performed using MS-Excel 2017.

## 5. Conclusions

**Figure 12:**
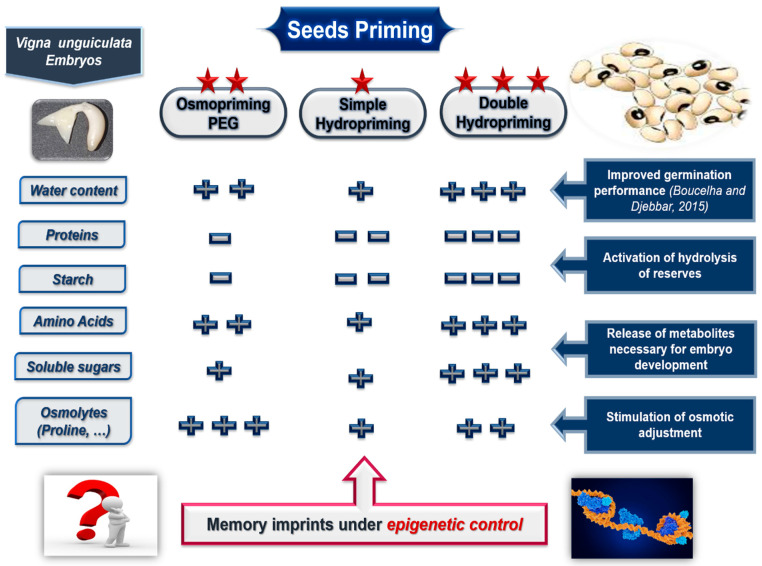
Graphical abstract illustrating the effects of priming types on nitrogen and carbon metabolism in *Vigna unguiculata* embryos [2].

This work aimed to contribute to the understanding of the biochemical mechanisms governing seed priming at the embryonic level (cotyledons, radicles, and plumules). The results obtained within the framework of our experiments allow us to conclude that seeds priming causes quantitative and qualitative modifications at the levels of metabolites of the cotyledons and embryonic axes, via the activation of enzymes involved in the degradation of proteins and carbohydrate reserves, stimulation of the biosynthesis of osmolytes such as free proline and soluble sugars, the production of certain sugars and amino acids involved in the development of the embryo, vegetative growth, and the formation of the cellular skeleton of the new seedling. These biochemical modifications depend on the organ and the treatment method applied to the seeds, as a double cycle of hydration—dehydration results in the widest range of metabolic modifications (Figure 12). These findings reveal that the biochemical and physiological mechanisms involved in seed priming involve several complex and heterogeneous regulatory and signaling networks controlled by the expression of numerous genes. This study represents only the beginning of long-term work to better understand the mechanisms involved in seed priming and to answer certain questions that remain. A deeper understanding of the pre-germinative metabolism requires the identification of key genes regulating hydrolases, antioxidant enzymes, LEA proteins, osmoprotectants, signaling molecules, and hormonal balance.

## Figures and Tables

**Table 1:** Percentages of change in the different parameters studied compared to the control.

Parameters	Organ	*6 h Imbib*	*PEG 30%*	*3 h Hydro*	*6 h Hydro*	*3 h Double Hydro*
Water content	*Cotyledons*	+22.42%	+27%	+12.81%	+17.84%	+26%
	*Radicle*	+12.42%	+17.17%	+8.51%	+10.25%	+17.73%
	*Plumule*	+8.51%	+15.71%	+16.72%	+18.91%	+24.6%
Proteins content	*Cotyledons*	−8.12%	−13.17%	−15.88%	−27.7%	−34.85%
	*Radicle*	−7.73%	−9.93%	−13.83%	−21.54%	−31.75%
	*Plumule*	−4.47%	−15.74%	−18.22%	−33.32%	−45.75%
Amino acids content	*Cotyledons*	+6.96%	+96.72%	+59.89%	+110.49%	+200%
	*Radicle*	+14.8%	+80.95%	+27.94%	+48.53%	+144.83%
	*Plumule*	+9.19%	+71.1%	+53.39%	+85.84%	+176.55%
Amino acid/proteins ratio	*Cotyledons*	+14.45%	+122.75%	+86.87%	+183.9%	+352.04%
	*Radicle*	+24.51%	+101.06%	+48.65%	+89.46%	+259%
	*Plumule*	+13.91%	+102.36%	+86.92%	+177.73%	+408%
Free proline content	*Cotyledons*	−61%	+57.58%	+21.88%	+51.09%	+63.72%
	*Radicle*	−78.97%	+83.33%	+10.22%	+72.22%	+105.44%
	*Plumule*	−16.7%	+702%	+228.10%	+306.25%	+490%
Free proline/ total amino acids ratio	*Cotyledons*	−63.74%	−19.75%	−24.07%	−27.78%	−45.24%
	*Radicle*	−81.96%	−0.18%	−8.62%	−10.34%	−17.24%
	*Plumule*	−25%	+306.92%	+110.32%	+100%	+60%
Starch content	*Cotyledons*	−22.5%	−35.71%	−32.5%	−36.67%	−40%
	*Radicle*	−12.34%	−32.74%	−25.62%	−37.55%	−46.61%
	*Plumule*	−9.11%	−15.85%	−12.23%	−27.14%	−40.35%
Soluble sugars content	*Cotyledons*	+27.13%	+57.84%	+44.05%	+60.86%	+91.4%
	*Radicle*	+9.74%	+32.02%	+28%	+34.41%	+42.22%
	*Plumule*	+3.8%	+12.51%	+5.92%	+21.22%	+60.2%
Soluble sugars/starch ratio	*Cotyledons*	+66.27%	+148.86%	+116.30%	+157.15%	+223.33%
	*Radicle*	+24.89%	+96.29%	+72.08%	+115.24%	+166.39%
	*Plumule*	+14.24%	+33.75%	+20.72%	+66.43%	+168.64%

**Table 2:** Composition of amino acids present in the three organs of *Vigna unguiculata* seeds according to the different treatments. The symbol (-) indicates the absence of the amino acid, while the symbol (+) indicates its presence in the organ. The number of (+) marks represents the intensity of the stain revealed on the TLC plate.

Amino Acids	Organ	*Control*	*6 h Imbib*	*PEG 30%*	*3 h Hydro*	*6 h Hydro*	*3 h Double Hydro*
Alanine	*Radicle*	+	+	+	+	+	++
Arginine	*Plumule*	+	+	+	+	+	++
Aspartate	*Radicle*	+	+	+	+	+	+
Cysteine	*Cotyledons*	+	+	+	+	+	+
	*Radicle*	-	-	-	-	-	+
	*Plumule*	-	-	-	-	-	+
Glycine	*Cotyledons*	+	+	+	+	+	++
Histidine	*Radicle*	+	+	+	+	+	+
Isoleucine	*Radicle*	-	-	+	+	+	++
Methionine	*Radicle*	+	+	+	+	+	++
Ornithine	*Cotyledons*	+	+	+	+	+	++
Proline	*Cotyledons*	+	+	+++	+	+	++
	*Radicle*	+	+	++	+	+	+++
	*Plumule*	+	+	+++	+	+	++
Serine	*Plumule*	-	-	-	-	-	+
Threonine	*Cotyledons*	-	-	-	-	+	++
	*Radicle*	+	+	+	+	+	++
	*Plumule*	+	+	+	+	+	++
Tryptophane	*Radicle*	+	+	+	+	+	++
	*Plumule*	-	-	-	-	-	+
Valine	*Cotyledons*	-	-	-	-	-	+
	*Radicle*	+	+	+	+	+	++
	*Plumule*	+	+	+	+	+	++

**Table 3:** Composition of soluble sugars in the three organs of *Vigna unguiculata* seeds according to the different treatments. The symbol (-) indicates the absence of the sugar, while the symbol (+) indicates its presence in the organ. The number of (+) marks represents the intensity of the stain revealed on the TLC plate.

Soluble Sugars	Organ	*Control*	*6 h Imbib*	*PEG 30%*	*3 h Hydro*	*6 h Hydro*	*3 h Double Hydro*
Gluconic Acid	*Cotyledons*	++	++	+	+	+	+
	*Radicle*	++	++	++	++	+	+
	*Plumule*	-	+	++	++	++	+++
Arabinose	*Plumule*	-	-	-	-	-	+
Fructose	*Cotyledons*	-	-	-	-	-	+
	*Radicle*	+	+	+	+	+	+
Galactose	*Cotyledons*	++	++	+	++	++	+
	*Radicle*	+	+	+	+	+	++
Glucose	*Radicle*	-	-	-	-	-	+
	*Plumule*	+	+	+	+	+	++
Maltose	*Cotyledons*	+	+	-	+	+	-
	*Radicle*	+	+	+	+	+	+
	*Plumule*	-	+	++	++	++	+++
Sucrose	*Cotyledons*	-	-	-	-	+	++
Rhamnose	*Plumule*	-	-	-	-	-	+
Ribose	*Cotyledons*	-	-	-	-	-	+
Trehalose	*Plumule*	-	-	-	-	+	++
Xylose	*Radicle*	-	-	-	-	-	+

## Data Availability

Data will be made available upon request.
